# A randomized, double-blind, placebo-controlled study of vortioxetine on cognitive function in depressed adults

**DOI:** 10.1017/S1461145714000546

**Published:** 2014-04-30

**Authors:** Roger S. McIntyre, Søren Lophaven, Christina K. Olsen

**Affiliations:** 1 Mood Disorders Psychopharmacology Unit, University Health Network, University of Toronto, Toronto, ON, Canada; 2 H. Lundbeck A/S, Copenhagen, Denmark

**Keywords:** Cognitive function, path analysis, recurrent major depression, tolerability

## Abstract

The efficacy of vortioxetine 10 and 20 mg/d *vs.* placebo on cognitive function and depression in adults with recurrent moderate-to-severe major depressive disorder (MDD) was evaluated. Patients (18–65 yr, *N* = 602) were randomized (1:1:1) to vortioxetine 10 or 20 mg/d or placebo for 8 wk in a double-blind multi-national study. Cognitive function was assessed with objective neuropsychological tests of executive function, processing speed, attention and learning and memory, and a subjective cognitive measure. The primary outcome measure was change from baseline to week 8 in a composite z-score comprising the Digit Symbol Substitution Test (DSST) and Rey Auditory Verbal Learning Test (RAVLT) scores. Depressive symptoms were assessed using the Montgomery-Åsberg Depression Rating Scale (MADRS). In the pre-defined primary efficacy analysis, both doses of vortioxetine were significantly better than placebo, with mean treatment differences *vs.* placebo of 0.36 (vortioxetine 10 mg, *p* < 0.0001) and 0.33 (vortioxetine 20 mg, *p* < 0.0001) on the composite cognition score. Significant improvement *vs.* placebo was observed for vortioxetine on most of the secondary objectives and subjective patient-reported cognitive measures. The differences to placebo in the MADRS total score at week 8 were −4.7 (10 mg: *p* < 0.0001) and −6.7 (20 mg: *p* < 0.0001). Path and subgroup analyses indicate that the beneficial effect of vortioxetine on cognition is largely a direct treatment effect. No safety concern emerged with vortioxetine. Vortioxetine significantly improved objective and subjective measures of cognitive function in adults with recurrent MDD and these effects were largely independent of its effect on improving depressive symptoms.

## Introduction

Major depressive disorder (MDD) is a common mental disorder often associated with deficits in cognitive function (see McIntyre et al., 2013 for a recent review). The Diagnostic and Statistical Manual 5 (DSM-5) lists impairment in cognition (i.e. diminished ability to think or concentrate or indecisiveness) as a criterion item in the diagnosis of a major depressive episode (MDE). In addition to being a reason for frequent subjective complaints, objective deficits in measures of executive function, processing speed, attention, learning and memory during, and after resolution of an MDE have been reported (Porter et al., 2007; Hammar and Ardal, 2009; Baune et al., 2010).

The estimated annual costs attributable to MDD are $83 billion in the US, with indirect costs due to decreased psychosocial function (notably workforce performance) being a major contributor (Greenberg et al., 2003). Preliminary evidence suggests that cognitive dysfunction is an important mediator of functional impairment (i.e. workplace performance) in individuals with MDD (Buist-Bouwman et al., 2008). Moreover, it has been proposed that improvement in cognitive function significantly influences functional recovery from an MDE (Jaeger et al., 2006; Greer et al., 2010).

Vortioxetine (1-[2-(2,4-dimethyl-phenylsulfanyl)-phenyl]-piperazine-hydrobromide, Lu AA21004) is a novel antidepressant that has demonstrated efficacy in doses up to 20 mg/d in short-term studies of 6–8 wk duration in adult patients with MDD (Alvarez et al., 2012; Henigsberg et al., 2012; Boulenger et al., 2014). Its principal mode of action is hypothesized to occur via the combination of a direct effect on receptor activity and serotonin (5-HT) reuptake inhibition (Bang-Andersen et al., 2011; Westrich et al., 2012). *In vitro* studies in recombinant cell lines show that vortioxetine is a 5-HT_3_, 5-HT_1D_, and 5-HT_7_ receptor antagonist, 5-HT_1B_ receptor partial agonist, 5-HT_1A_ receptor agonist, and a 5-HT transporter inhibitor (Bang-Andersen et al., 2011; Mørk et al., 2012; Westrich et al., 2012).

The efficacy of vortioxetine 5 mg/d on both depressive symptoms and cognitive function has been demonstrated in a placebo-controlled 8 wk study with duloxetine as active reference in patients aged ⩾65 yr with MDD (Katona et al., 2012). That study was designed to compare the effect of vortioxetine to that of placebo on depressive symptom severity, with cognitive function as a secondary efficacy outcome.

To our knowledge, only one large study has primarily aimed to compare the efficacy of a conventional antidepressant *vs.* placebo on cognition (Raskin et al., 2007). That study reported that duloxetine demonstrated significantly greater improvement in a composite cognitive score (mediated largely by improvement in verbal learning and memory) than placebo in elderly patients (aged ⩾65 yr) with recurrent MDD. Studies are challenged by the heterogeneity of cognitive dysfunction and few studies have evaluated the effect of antidepressants on non-emotional, objectively measured cognitive performance in non-elderly, adults with MDD, and they generally have a small sample size, are not placebo-controlled and assess treatment effect compared to baseline (Biringer et al., 2009; McIntyre et al., 2013).

Herein, we primarily aimed to evaluate the efficacy of vortioxetine 10 and 20 mg/d *vs.* placebo on cognitive function in adults (aged ⩽65 yr) with recurrent MDD during a depressive episode of moderate severity or greater. The primary efficacy endpoint was a composite cognition score comprising the Digit Symbol Substitution Test (DSST) and the Rey Auditory Verbal Learning Test (RAVLT). These and homologous tests address key cognitive domains shown to be impaired in patients with depression and have demonstrated clinical sensitivity in MDD in the elderly population (Raskin et al., 2007; Katona et al., 2012). The present study aimed to extend the investigation to the adult MDD population including a broader assessment of objective and subjective measures of cognition in addition to depressive symptoms, safety and tolerability as secondary outcomes.

## Method

### Patients

This double-blind, randomized, fixed-dose, placebo-controlled, study included patients from 79 psychiatric inpatient and outpatient settings in 12 countries (Australia, Canada, Finland, France, Germany, Latvia, Mexico, Serbia, Slovakia, South Africa, Ukraine, and the USA) from December 2011 to May 2013. Patients were recruited by advertisements in 9 countries (Australia, Canada, Finland, France, Germany, Latvia, Mexico, South Africa, and the USA) or via referrals from general practitioners. The study was conducted in accordance with the principles of *Good Clinical Practice* (ICH, 1996) and the *Declaration of Helsinki* (WMA, 2008). Local research ethics committees approved the trial design, and all eligible patients provided written informed consent before participating.

### Randomization and treatment

Eligible patients were assigned to double-blind treatment according to a randomization list that was computer generated by H. Lundbeck A/S. The details of the randomization series were contained in a set of sealed opaque envelopes. At each site, sequentially enrolled patients were assigned the lowest randomization number available in blocks of 6 using an interactive voice/web response system. All investigators, trial personnel and patients were blinded to treatment assignment for the duration of the study. The randomization code was not broken for any patient during the study.

### Study procedures

After a screening period of up to 10 d, 602 eligible patients were randomized (1:1:1) to vortioxetine 10 mg/d, vortioxetine 20 mg/d, or placebo for 8 wk of double-blind treatment. Patients in the vortioxetine 20 mg/d group received vortioxetine 10 mg/d in week 1 and 20 mg/d from weeks 2 to 8. Patients were seen at baseline, and weeks 1, 4, and 8. Patients who withdrew prior to study completion were evaluated at the earliest possible date after withdrawal. A safety follow-up contact was scheduled for 4 wk after completion of the treatment period or after withdrawal from the study. Study medications were given as capsules of identical appearance. Following randomization, patients were instructed to take one capsule per day, orally, preferably in the morning.

Patients aged ⩾18 and ⩽65 yr, with a primary diagnosis of recurrent MDD according to DSM IV-TR criteria, a current MDE ⩾3 months’ duration (confirmed using the Mini International Neuropsychiatric Interview (Lecrubier et al., 1997)) and a Montgomery-Åsberg Depression Rating Scale (MADRS) total score ⩾26 at screening and baseline visits were eligible for inclusion in the study. Patient inclusion and exclusion criteria for this study were similar to other studies in MDD. For patient exclusion criteria, see the Supplementary Material. In brief, patients were excluded if they were taking psychotropic drugs within 2 wk prior to baseline or during the study, including reversible or irreversible monoamine oxidase inhibitors, serotonin reuptake inhibitors (fluoxetine within 5 wk), serotonin noradrenaline reuptake inhibitors, tricyclic antidepressants, psychoactive herbal remedies, any drug used for augmentation of antidepressant action or any other antidepressant drugs, oral antipsychotic and anti-manic drugs, or dopamine antagonists, or any anxiolytics.

### Outcome measures

The effect of vortioxetine (10 or 20 mg/d) *vs.* placebo on cognitive function was assessed using the following neuropsychological tests: DSST (executive function, speed of processing, attention), RAVLT (learning, memory), Trail Making Test A/B (TMT-A: speed of processing; TMT-B: executive function), Stroop test (congruent and incongruent: executive function); Simple reaction time task (SRT: speed of processing), and the Choice reaction time task (CRT: attention) at baseline, week 1 and week 8. In addition, the patient-reported cognitive measure, Perceived Deficits Questionnaire (PDQ) comprising four subscales: attention/concentration, prospective memory, planning/organization, and retrospective memory was assessed at baseline and week 8. For details of these assessments, see the Supplementary Material.

The MADRS and Clinical Global Impression – Severity of Illness (CGI-S) were assessed at baseline, and weeks 1, 4, and 8, and the Clinical Global Impression – Global Improvement (CGI-I) at weeks 1, 4, and 8. The MADRS was administered after the neuropsychological tests and before the Columbia-Suicide Severity Rating Scale (C-SSRS) and CGI scales.

### Statistical analysis

Safety analyses were based on the *all-patients-treated set* (APTS), comprising all randomized patients who took at least one dose of study medication. Efficacy analyses were based on a modified *intention-to-treat set* – the *full-analysis set* (FAS), comprising all patients in the APTS who had at least one valid post-baseline assessment of the primary efficacy variable (the DSST and the RAVLT [acquisition and delayed recall]).

The primary efficacy analysis was the change from baseline to week 8 in the composite z-score defined as the equally weighted sum of the z-scores in the DSST and RAVLT, thus assessing a broad range of cognitive domains, including executive function, attention, processing speed, and learning and memory. The DSST score was assigned a weight of 0.5, and the two subtest scores of the RAVLT (acquisition [learning] and delayed recall [memory]) were each assigned a weight of 0.25. The composite z-score is used for the first time in this study and is based on *post-hoc* analysis of the vortioxetine study of elderly patients with MDD (Katona et al., 2012). Based on a Missing-at-Random assumption, these analyses were performed using all available data from all patients in the FAS. The model included treatment and center as fixed factors. The baseline composite z-score was used as a covariate. Interactions between visit and treatment and baseline composite z-score were also included in the model. An unstructured covariance structure was used to model the within-patient variation. For endpoints that occurred after the pre-specified statistical testing procedure was stopped or that were outside the testing procedure, nominal p-values with no adjustment for multiplicity were reported. The phrasing ‘separation from placebo’ is used to describe findings with *p* < 0.05. Efficacy analyses that were not multiplicity-controlled were considered secondary. For details of the testing hierarchy and descriptions of key secondary and secondary analyses, multiple regression analyses [path analysis] and *post-hoc* sub-group analyses, see the Supplementary Material.

The sample size calculation was based on an overall significance level of 5% by having 2.5% within each dose in order to adjust for multiplicity. For the primary endpoint (composite z-score), the treatment difference to placebo for each vortioxetine dose at week 8 was assumed to be 0.25, based on the results with elderly patients (Katona et al., 2012). A total of 600 patients (200 per arm) were needed for the mixed model for repeated measures (MMRM) using all available data to provide a power of ≈90% for finding at least one dose significant, and a power of ≈85% for finding a specific dose significant, assuming a 20% withdrawal rate.

### Safety and tolerability assessments

Starting at baseline, patients were asked a non-leading question at each visit (such as, ‘*how do you feel?’*). All adverse events (AEs), either observed by the investigator or reported spontaneously by the patient, were recorded together with vital signs. Qualified personnel coded AEs using the lowest level term according to MedDRA, version 15.1. The incidence of individual AEs was compared between treatment groups using Fisher's exact test. Clinical safety laboratory tests, weight, body mass index (BMI), electrocardiographs (ECGs), and physical examination findings were also evaluated. Potential relationships between study drug and suicidality were assessed using the C-SSRS (US FDA, 2010). As a *post-hoc* analysis, the safety database was searched at the verbatim (investigator's term) level for possible suicide-related AEs (Laughren, 2006).

## Results

### Study sample

The APTS included 598 patients after the exclusion of 4 patients who did not take any study medication (see Supplementary Material, Fig. S1). With a mean baseline MADRS total score of 31.6, patients were moderately to severely depressed. There were no clinically relevant differences between treatment groups in demographic or clinical characteristics at baseline (Table 1).

**Table 1.** Demographics and baseline clinical characteristics (APTS)

	Placebo (*n* = 196)	Vortioxetine 10 mg (*n* = 195)	Vortioxetine 20 mg (*n* = 207)
Women *n* (%)	129 (65.8%)	134 (68.7%)	133 (64.3%)
Mean age ± s.d. (yr)	45.6 ± 12.1	45.4 ± 12.2	46.1 ± 11.8
Range (yr)	19–65	18–65	18–65
Caucasian (%)	95.9%	93.8%	93.7%
Median length of current MDE (wk)	18	19	19
Previous MDEs, mean ± s.d. (*n*)	2.4 ± 2.0	2.3 ± 1.7	2.6 ± 2.1
Range (*n*)	1–11	1–11	1–13
Assessment scores (FAS), mean ± s.d.	(*n* = 194)	(*n* = 193)	(*n* = 204)
MADRS total score	31.3 ± 3.8	31.6 ± 3.8	31.7 ± 3.5
CGI-S	4.55 ± 0.63	4.60 ± 0.62	4.62 ± 0.58
DSST_correct symbols_	42.4 ± 14	42.0 ± 13	41.6 ± 13
RAVLT_acquisition_	22.1 ± 6	22.3 ± 6	22.6 ± 6
RAVLT_delayed recall_	5.70 ± 2.8	5.76 ± 2.8	6.05 ± 3.1
PDQ_total score_	39.8 ± 12	41.4 ± 12	41.1 ± 12
PDQ_attention/concentration_	11.9 ± 3.3	12.4 ± 3.4	12.4 ± 3.2
PDQ_prospective memory_	7.32 ± 3.2	7.85 ± 3.3	7.61 ± 3.4
PDQ_planning/organization_	11.6 ± 3.7	11.6 ± 3.7	11.8 ± 3.9
PDQ_retrospective memory_	8.98 ± 3.8	9.53 ± 3.6	9.28 ± 4.0
TMT-A (s)	48.7 ± 25	46.5 ± 24	46.2 ± 27
TMT-B (s)	105 ± 53	102 ± 52	103 ± 52
Stroop_congruent_ (s)	50.0 ± 25	49.6 ± 25	50.0 ± 28
Stroop_incongruent_ (s)	85.7 ± 39	85.0 ± 41	83.6 ± 41
SRT (log_10_ ms)	2.64 ± 0.20	2.64 ± 0.20	2.63 ± 0.20
CRT (log_10_ ms)	2.78 ± 0.14	2.78 ± 0.14	2.78 ± 0.14

APTS, all patients treated set; CGI-S, Clinical Global Impression – Severity; CRT, choice reaction time task; DSST, Digit Symbol Substitution Test; FAS, full-analysis set; MADRS, Montgomery-Åsberg Depression Rating Scale; MDE, major depressive episode; PDQ, Perceived Deficits Questionnaire; RAVLT, Rey Auditory Verbal Learning Test; s.d., standard deviation; SRT, simple reaction time task; TMT, trail making test.

### Withdrawals from the study

There were no differences to placebo in either of the active treatment groups in the proportion of patients who withdrew from the study. The proportions of patients who withdrew during treatment because of treatment-emergent AEs were 4.1% (placebo), 2.6% (vortioxetine 10 mg) and 4.3% (vortioxetine 20 mg). Approximately 87% of the patients in each group received study medication for 50–63 d. The total exposure accrued in each treatment group was approximately 28 patient-yr.

## Efficacy

### Primary efficacy endpoint

In the pre-defined primary efficacy analysis, both doses of vortioxetine were significantly superior *vs.* placebo in mean change from baseline to week 8 in the composite z-score (FAS, MMRM), with a mean treatment difference to placebo of 0.36 [95% CI: 0.22;0.50] (vortioxetine 10 mg, *p* < 0.0001) and 0.33 [95% CI: 0.19; 0.47] (vortioxetine 20 mg, *p* < 0.0001) (Table 2).

**Table 2.** Efficacy analyses, change from baseline to week 8, difference to placebo (mean±s.e. [95% CI]) (FAS, MMRM)

	Placebo (*n* = 194)	Vortioxetine 10 mg (*n* = 193)			Vortioxetine 20 mg (*n* = 204)		
	Δ baseline	Δ baseline	Δ placebo	*p*-value	Δ baseline	Δ placebo	*p*-value
Primary endpoint							
Composite z- score (DSST/RAVLT_acq_/RAVLT_delay_)	−0.24 ± 0.05^a^	0.13 ± 0.05	0.36 ± 0.07 [0.22; 0.50]	<0.001	0.10 ± 0.05	0.33 ± 0.07 [0.19; 0.47]	<0.001
Key secondary endpoints							
DSST_correct symbols_	4.83 ± 0.63	9.03 ± 0.63	4.20 ± 0.87 [2.50; 5.90]	<0.001	9.09 ± 0.61	4.26 ± 0.86 [2.57; 5.94]	<0.001
RAVLT_acquisition_	3.06 ± 0.34	4.08 ± 0.34	1.02 ± 0.46 [0.11; 1.93]	0.029	3.65 ± 0.33	0.59 ± 0.46 [−0.31; 1.50]	0.199
RAVLT_delayed recall_	0.91 ± 0.18	1.63 ± 0.18	0.71 ± 0.24 [0.24; 1.19]	0.003	1.56 ± 0.17	0.65 ± 0.24 [0.17; 1.12]	0.007
Secondary cognition endpoints							
TMT-A (s)	−7.1 ± 1.0	−10.8 ± 1.0	−3.8 ± 1.4 [−6.4; −1.1]	0.006	−10.9 ± 1.0	−3.8 ± 1.4 [−6.5; −1.1]	0.005
TMT-B (s)	−13.8 ± 2.0	−21.4 ± 2.0	−7.6 ± 2.7 [−12.9; −2.2]	0.006	−22.8 ± 1.9	−9.0 ± 2.7 [−14.3; −3.7]	<0.001
SRT (log_10_ ms)	−0.007 ± 0.009	−0.053 ± 0.009	−0.046 ± 0.012 [−0.069; −0.022]	<0.001	−0.037 ± 0.009	−0.029 ± 0.012 [−0.053; −0.0055]	0.016
CRT (log_10_ ms)	−0.015 ± 0.007	−0.046 ± 0.007	−0.032 ± 0.009 [−0.049; −0.014]	<0.001	−0.023 ± 0.006	−0.008 ± 0.009 [−0.026; 0.0093]	0.355
Stroop_congruent_ (s)	−6.0 ± 0.9	−10.0 ± 0.9	−4.0 ± 1.3 [−6.5; −1.5]	0.002	−10.4 ± 0.9	−4.5 ± 1.3 [−6.9; −2.0]	<0.001
Stroop_incongruent_ (s)	−10.9 ± 1.5	−17.7 ± 1.5	−6.8 ± 2.0 [−10.8; −2.7]	0.001	−17.5 ± 1.4	−6.5 ± 2.0 [−10.5; −2.5]	0.001
PDQ_total score_*	−7.8 ± 0.9	−12.2 ± 0.9	−4.4 ± 1.2 [−6.8; −2.1]	<0.001	−13.5 ± 0.9	−5.7 ± 1.2 [−8.0; −3.4]	<0.001
PDQ_attention/concentration_*	−2.2 ± 0.3	−3.7 ± 0.3	−1.5 ± 0.4 [−2.2; −0.8]	<0.001	−4.1 ± 0.3	−1.9 ± 0.4 [−2.6; −1.2]	<0.001
PDQ_prospective memory_*	−1.7 ± 0.2	−2.4 ± 0.2	−0.8 ± 0.3 [−1.3; −0.2]	0.006	−2.5 ± 0.2	−0.8 ± 0.3 [−1.4; −0.3]	0.003
PDQ_planning/organization_*	−2.3 ± 0.3	−3.3 ± 0.3	−1.0 ± 0.4 [−1.8; −0.2]	0.012	−3.9 ± 0.3	−1.6 ± 0.4 [−2.3; −0.8]	<0.001
PDQ_retrospective memory_*	−1.7 ± 0.3	−2.6 ± 0.3	−1.0 ± 0.3 [−1.6; −0.3]	0.004	−3.0 ± 0.2	−1.3 ± 0.3 [−2.0; −0.7]	<0.001
Secondary depression efficacy variables							
MADRS total score	−10.9 ± 0.6	−15.6 ± 0.6	−4.7 ± 0.9 [−6.4; −3.0]	<0.001	−17.6 ± 0.6	−6.7 ± 0.9 [−8.4; −5.0]	<0.001
CGI-I score^b^	2.85 ± 0.08	2.24 ± 0.08	−0.61 ± 0.11 [−0.81; −0.40]	<0.001	1.99 ± 0.07	−0.86 ± 0.11 [−1.1; −0.65]	<0.001
CGI-S score	−1.15 ± 0.08	−1.80 ± 0.08	−0.65 ± 0.12 [−0.88; −0.42]	<0.001	−2.00 ± 0.08	−0.85 ± 0.12 [−1.1; −0.62]	<0.001
MADRS response (FAS, LOCF)^b^	29.4%	47.7%	–	<0.001	58.8%	–	<0.001
MADRS remission (FAS, LOCF)^b^	17.0%	29.5%	–	0.003	38.2%	–	<0.001
CGI-I response (CGI-I ⩽2)^b^	38.7%	61.7%	–	<0.001	70.1%	–	<0.001
CGI-S remission (CGI-S ⩽2)^b^	19.6%	37.8%	–	<0.001	42.6%	–	<0.001

a The negative mean composite z-score for placebo indicates that patients on placebo perform worse than average on cognition, i.e. it does not indicate that these patients deteriorated during treatment.

b Absolute value. *FAS, ANCOVA, LOCF. ANCOVA: analysis of covariance, CGI-I, Clinical Global Impression – Improvement; CGI-S, Clinical Global Impression – Severity; CI, confidence interval; CRT, choice reaction time task; DSST, Digit Symbol Substitution Test; FAS, full-analysis set; LOCF, last observation carried forward; MADRS, Montgomery-Åsberg Depression Rating Scale; MMRM, mixed model for repeated measures; OC, observed cases; PDQ, Perceived Deficits Questionnaire; RAVLT, Rey Auditory Verbal Learning Test; SRT, simple reaction time task; TMT, trail making task.

### Key secondary efficacy endpoints

Both doses of vortioxetine were significantly superior to placebo in the pre-defined key secondary efficacy analysis of the DSST score (Table 2). For the RAVLT (acquisition), the *p*-value for each dose was >0.025, and the testing sequence stopped at this point. However, for the RAVLT (delayed recall), the *p*-value for each dose was <0.025.

### Secondary efficacy endpoints

#### Cognitive function

**Fig. 1.**
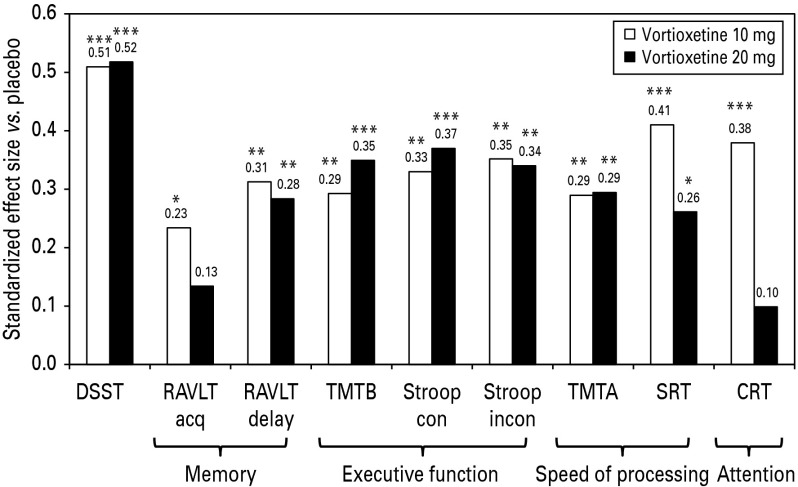
Standardized effect size (Cohen's *d*) for the neuropsychological tests (FAS, OC). DSST, Digit Symbol Substitution Test; FAS, full-analysis set; OC, observed cases; RAVLT, Rey Auditory Verbal Learning Test; TMT, Trail Making Test, Stroop; SRT, simple reaction time task; CRT, choice reaction time task. *p < 0.05, **p < 0.01, ***p < 0.001 *vs.* placebo. p-values for TMT, Stroop, SRT and CRT are not corrected for multiplicity.

At week 8, separation from placebo (*p* < 0.05) was seen for all other measures of cognitive function (TMT-A/B; Stroop [congruent and incongruent]; SRT and CRT), with the exception of CRT for vortioxetine 20 mg (Table 2). In *post-hoc* analyses, standardized effect sizes (Cohen's *d*) for the neuropsychological tests (FAS, OC) in which *p* < 0.05 were 0.51 and 0.52 (DSST), 0.23 (RAVLT [acquisition] 10 mg), 0.31 and 0.28 (RAVLT [delayed recall]), 0.29 and 0.35 (TMT-B), 0.33 and 0.37 (Stroop congruent), 0.35 and 0.34 (Stroop incongruent), 0.29 and 0.29 (TMT-A), 0.41 and 0.26 (SRT), and 0.38 (CRT 10 mg) for vortioxetine 10 and 20 mg, respectively (Fig. 1). On the patient-rated PDQ total score and on PDQ subscale scores at week 8, patients in both vortioxetine groups separated from placebo (*p* < 0.05) (Table 2).

#### Depressive symptoms

**Fig. 2.**
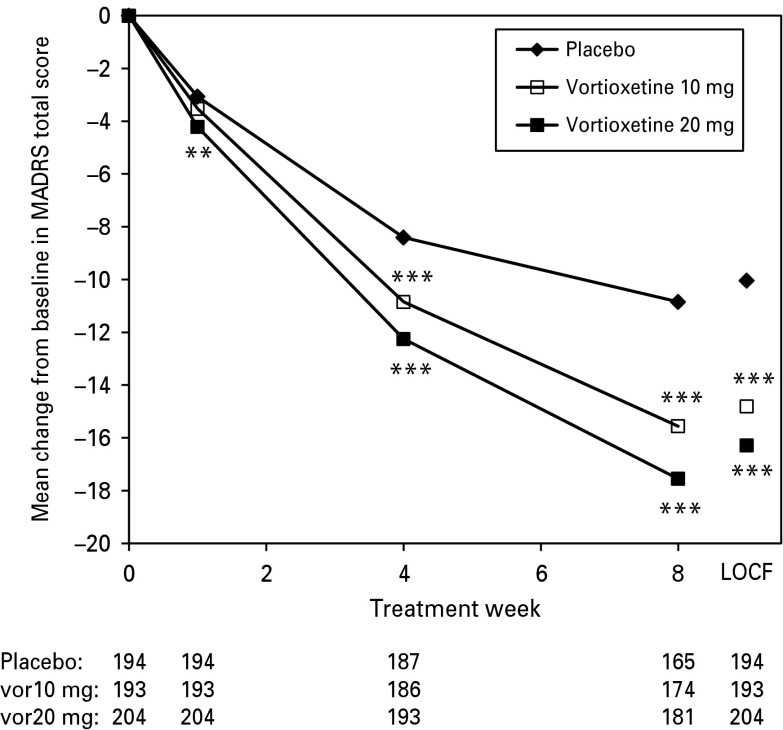
Estimated Montgomery-Åsberg Depression Rating Scale (MADRS) total scores from baseline to week 8 (FAS, MMRM by visit) and LOCF, FAS, ANCOVA). The mean improvement from baseline to week 8 in the MADRS total score was −10.9 points (placebo), −15.6 points (vortioxetine 10 mg), and −17.6 points (vortioxetine 20 mg). ANCOVA, analysis of covariance; FAS, full-analysis set; LOCF, last observation carried forward; MMRM, mixed model for repeated measures. Patient numbers at each visit are shown below the *x*-axis for each treatment group. **p < 0.01; ***p < 0.001 *vs.* placebo. *p*-values are not corrected for multiplicity.

Patients in both vortioxetine groups separated from placebo in depressive symptom and CGI variables [MADRS total score, CGI-S score, CGI-I score, and response (⩾50% reduction from baseline in MADRS total score or a CGI-I score ⩽2) and remission (MADRS total score ⩽10 or a CGI-S score ⩽2) rates] (Table 2). Separation from placebo in the change from baseline in MADRS total score was seen from week 1 onward for vortioxetine 20 mg and from week 4 onward for vortioxetine 10 mg, with greater improvement at the higher dose (Fig. 2). Similar results were found using analysis of covariance (ANCOVA) [observed cases (OC) and last observation carried forward (LOCF)].

#### Separation of effect on cognitive performance and depressive symptoms

After correction for the effect on MADRS, both vortioxetine doses improved cognitive performance, indicating an effect on cognitive function independent of its effect on improving depressive symptoms. At week 8, with the MADRS total score as the depression mediator, the pre-specified multiple regression analyses (path analysis) based on the FAS (ANCOVA, LOCF) showed a significant direct effect on the composite z-score (primary endpoint) and on the mean difference to placebo in DSST score (key secondary endpoint), which was the main driver in the primary analysis (Table 3).

**Table 3.** Change from baseline to week 8 in composite z-score and DSST (number of correct symbols) – depression mediator: change from baseline in Montgomery-Åsberg Depression Rating Scale (MADRS) total score

Variable	Vortioxetine 10 mg	Vortioxetine 20 mg
	Difference to placebo at week 8 (FAS, LOCF)	
All patients	*n* = 193	*n* = 204
Effect on composite z-score after correcting for effect on MADRS	0.23***	0.15*
Effect on DSST after correction for effect on MADRS	2.59**	2.23**
	Direct treatment effect: % (95% CI) (FAS, LOCF)	
Composite z-score	64 (47; 82)	48 (23; 73)
DSST_correct symbols_	66 (47; 84)	56 (34; 78)
	Difference to placebo at week 8 (FAS, MMRM)	
Non-responders^§^	*n* = 92	*n* = 68
Composite z-score	0.20*	0.28**
DSST_correct symbols_	2.25*	2.88**
Non-remitters (MADRS > 10)	*n* = 123	*n* = 110
Composite z-score	0.26**	0.28**
DSST_correct symbols_	3.05**	3.53***

* *p* < 0.05, **p < 0.01, ***p < 0.001 *vs.* placebo.

§ <50% reduction from baseline to week 8 in MADRS total score.

The results of multiple regression analyses (path analysis) indicate that one-half to two-thirds of the treatment effect was a direct effect on cognitive performance (composite z-score and DSST) at week 8 (Table 3).

In addition, the dissociation between improvement in cognitive function and improvement of depressive symptoms was further supported by *post-hoc* analyses indicating that both vortioxetine doses improved cognitive performance (composite z-score and DSST) at week 8 in the subgroup of patients who were non-responders and the subgroup of patients who were non-remitters (Table 3).

The direct effects on the primary endpoint were in general supported by the direct effects estimated for the secondary neuropsychological tests. For Stroop incongruent and congruent, the direct effects were 70 and 84%, respectively, for vortioxetine 10 mg *vs.* placebo, and 58 and 80%, respectively, for vortioxetine 20 mg *vs.* placebo. For TMT-A and B the direct effects were 60 and 71%, respectively, for vortioxetine 10 mg *vs.* placebo, and 51 and 67%, respectively, for vortioxetine 20 mg *vs.* placebo. For SRT and CRT, the direct effects were 66 and 67%, respectively, for vortioxetine 10 mg *vs.* placebo. For SRT the direct effect was 26% for vortioxetine 20 mg *vs.* placebo. For CRT, vortioxetine 20 mg *vs.* placebo, the estimation of the direct effect was not possible, as the treatment effects with and without correcting for the change from baseline to week 8 in the MADRS total score had different signs.

## Safety and tolerability

### Treatment-emergent adverse events (TEAEs)

During the 8 wk treatment period, the proportions of patients with TEAEs were 38.3% (placebo: *n* = 75), 46.2% (vortioxetine 10 mg; *n* = 90) and 52.7% (vortioxetine 20 mg; *n* = 109). Common AEs (incidence ⩾5% for vortioxetine) were nausea (4.1, 16.4, 20.8%) and headache (7.1, 8.2, 12.6%) for placebo, vortioxetine 10 mg and vortioxetine 20 mg, respectively. During treatment, 22 patients withdrew because of TEAEs (see Supplementary Material, Fig. S1). TEAEs leading to withdrawal of ⩾2 patients were nausea (1.9%) and headache (1.0%), (vortioxetine 20 mg), and disturbance in attention (1.0%) and depression (1.0%) (placebo).

No TEAEs related to suicide or self-harm were found. The C-SSRS data showed no clinically relevant differences between groups at screening or during the study. None of the patients had suicidal behavior during treatment and the proportions of patients with suicidal ideation were 11% (placebo), 9.3% (vortioxetine 10 mg) and 13% (vortioxetine 20 mg). Improved scores from baseline to week 8 for MADRS item 10 (suicidal thoughts) were seen in all treatment groups.

Serious AEs were reported by 4 patients, 2 patients in the placebo group (cholecystitis and hiatus hernia) and 2 patients in the vortioxetine 20 mg group (hypertension and type I diabetes). No deaths occurred during this study. Clinically relevant changes over time or differences between treatment groups were not observed in clinical laboratory test results, vital signs, weight, or ECG parameters.

## Discussion

Although antidepressants are suggested to improve cognitive function to some degree in patients with MDD, there is a lack of adequate and well-controlled studies to investigate this (Biringer et al., 2009; McIntyre et al., 2013). This is the first large placebo-controlled randomized study to report a statistically significant improvement in objectively measured cognitive performance in adult patients (aged ⩽65 yr) with recurrent MDD, wherein the primary outcome measure evaluated multi-domains of cognitive function. In addition to demonstrating efficacy on a composite cognition score based on two tests covering several domains of relevance for patients with MDD, improvement with vortioxetine treatment was also noted on secondary objective and subjective measures of cognitive function. Improvement *vs.* placebo was seen on all included measures of executive function, attention, and processing speed, as well as with learning and memory.

The clinical relevance of the significant effect of vortioxetine on objective neuropsychological test scores was supported by the magnitude of the standardized effect sizes, which ranged from 0.23 to 0.52 [Cohen's *d*, Fig. 1)] where *p* < 0.05 and were above the clinically meaningful threshold of 0.2 (Cohen, 1988). The present study extended the evidence of the positive effect of vortioxetine on cognitive function previously demonstrated in patients aged ⩾65 yr with MDD (Katona et al., 2012), in which vortioxetine separated from placebo in both the DSST and the RAVLT with standardized effect sizes [(0.25 for the DSST, 0.27 for the RAVLT (acquisition) and 0.24 for the RAVLT (delayed recall)] a little lower than those found in the present study. The magnitude of the observed effect on cognitive dysfunction should be contextualized in studies in patients with MDD. The standardized effect size of the deficits seen in MDD patients is typically 0.2–0.6 below what would be normal, depending on the cognitive domain (Rund et al., 2006; Lee et al., 2012; Rock et al., 2013). For comparison, cognitive dysfunction in disorders such as Alzheimer's disease is greater than in MDD by several s.d., and the treatment effect with the commonly used cholinesterase inhibitors show standardized effect sizes of approximately 0.3 (Rockwood, 2004), although caution must be exercised when comparing different studies.

There is little evidence that current antidepressants improve cognitive function in patients with MDD independently of their effect on depressive symptoms. In the present study the efficacy of both doses of vortioxetine on cognitive function was largely a direct and independent effect, rather than an epiphenomenon of broad-based symptom improvement in depression. The direct effect is suggested by the results of the path and subgroup analyses. Path analysis, previously referred to as causal modeling, is an extension of a multiple linear regression with its own unique assumptions and conventions (Streiner, 2005). A positive effect on cognitive performance was observed for both doses of vortioxetine after correction for the alleviation of depressive symptoms. The proportions of direct effect were 64 and 48% for vortioxetine 10 and 20 mg, respectively for the composite z-score and 66 and 56%, respectively for the DSST score. This supports the previous findings revealing more than two-thirds of the effect on cognitive function is a direct effect (Katona et al., 2012). In addition, vortioxetine's direct effect is also suggested by improved cognitive function in the subset of patients who were either non-responders or non-remitters.

Subjectively experienced cognitive function and objective neuropsychological assessments in patients with affective disorders are not associated to a great extent (e.g. Svendsen et al., 2012; Naismith et al., 2007). In this study, in addition to demonstrating efficacy on disparate objective measures of cognitive function, vortioxetine treatment improved subjective measures of cognitive function, as assessed by the PDQ in the domains of prospective/retrospective memory, attention/concentration, and planning and organization. The result from this patient-rated assessment tool provides additional support for the clinical relevance of the observed treatment differences in the depressed population, as robust treatment differences were found both in objective and subjective tests.

Several expert guidelines posit that achieving remission from depressive symptoms should be the primary therapeutic objective in depression (Zimmerman et al., 2008). The definition of the remitted state is subject of debate, and often a cut-off on a symptom severity scale has been applied as a surrogate measure. Nonetheless, patients who meet such symptom-based definitions of remission often do not consider themselves in a remitted state, and a large proportion of these individuals report symptoms such as impaired concentration (Zimmerman et al., 2012). Since improvement in cognition significantly influences functional recovery from an MDE, it is important to objectively measure cognitive function in clinical studies. Moreover, surveys of outpatients with MDD indicate that the presence of positive mental health, return to one's normal self, and return to a premorbid level of functioning are the principal desired goals of treatment (Zimmerman et al., 2012), suggesting a role and a need for assessment tools complementary to the conventional symptom-based clinician-administered scales. In the present study, capturing the patient's perspective on their cognitive function as well as using objective neuropsychological tests showed improvement in cognitive function that was not captured by the MADRS, suggesting that these measures capture unique aspects of depression not otherwise addressed.

The 5-HT system not only plays a critical role in the regulation of mood, but is also intimately involved in the regulation of cognitive function, as evidenced by pre-clinical and clinical studies (e.g. Booij et al., 2005; Jensen et al., 2013). For example, available evidence indicates that several 5-HT receptor subtypes (e.g. 5-HT_1A_, 5-HT_1B_, 5-HT_2_, 5-HT_3_, 5-HT_4_, 5-HT_6_ and 5-HT_7_ receptors), including the receptor targets of vortioxetine, have the potential to modulate neurotransmitter systems that are essential for regulation of cognitive function (e.g. glutamate, acetylcholine, histamine, dopamine and noradrenaline [Mørk et al., 2013; Pehrson and Sanchez, 2013; Pehrson et al., 2013a]). In line with this, electroencephalographic studies in rats have indicated that vortioxetine activates cortical networks that are associated with cognitive processes and that 5-HT_1A_ receptor agonism and 5-HT_3_ and 5-HT_7_ receptor antagonism contribute to these activating effects of vortioxetine (Sanchez et al., 2012). Furthermore, *in vivo* and *in vitro* electrophysiology studies of vortioxetine have indicated that disinhibition of GABA interneurons plays an important role in the activation of the cortical and hippocampal networks involved in cognitive processes and that 5-HT_3_ receptor antagonism appears to play a key role (Dale et al., 2013; du Jardin et al., 2013; Mørk et al., 2013; Pehrson et al., 2013a, b). Finally, preclinical studies in animal models assessing the effect of vortioxetine on attention, learning and memory, and executive function (cognitive flexibility) indicate that vortioxetine has a profile distinct from those of other antidepressants (escitalopram and duloxetine) and the potential to improve cognitive function at doses associated with clinically relevant exposures (Sanchez et al., 2012; du Jardin et al., 2013; Mørk et al., 2013; Pehrson et al., 2013b).

In addition to demonstrating efficacy on cognitive function, both doses of vortioxetine had clinically meaningful effects on baseline-to-endpoint change in depressive symptom reduction, MADRS response and remission rates, as well as CGI measures. A dose-response in the present study showing a greater improvement of the higher dose of vortioxetine on the MADRS total score was not reflected on the cognitive performance scores, indicating that vortioxetine exerts its antidepressant and beneficial effect on cognitive function via a distinct mechanism. There is evidence that cognitive function varies independently of mood state in MDD. In particular, improvements in cognitive performance do not necessarily track improvement in mood symptoms, which may reflect the distinct neuronal basis of cognitive control and emotion regulation as it relates to depression (Harmer et al., 2002; Campbell and Macqueen, 2004; Murrough et al., 2011). The present study supports this, as an effect on cognitive function was shown to be independent of the effect on mood symptoms. It also raises the point that the effect on cognition and the patient's potential return to normal functioning may occur despite an inadequate response to mood symptoms, and this warrants further investigation.

The most commonly reported AEs (incidence ⩾5% for vortioxetine) were nausea and headache. The discontinuation rate due to AEs was similar to the rate for placebo, suggesting favorable tolerability profile. No safety concerns emerged for either dose of vortioxetine during this study.

Limitations of the present study include, but are not limited to; duration of treatment for only 8 wk; eligible patients may not be representative of adults with MDD who are seen in normal clinical practice; exclusion of individuals presenting with an index episode and/or milder baseline severity and our results only pertain to the two doses evaluated.

Notwithstanding, this appears to be the first large randomized study in adults (aged ⩽65 yr) to document a beneficial effect on a composite measure of cognitive function in MDD, wherein the pre-defined primary clinical outcome was cognitive performance as well as consistent improvement across a range of neuropsychological tests. The clinical relevance of the results herein is suggested by the magnitude of the standardized effect sizes and the improvements in patient-reported cognitive function. Future vistas could endeavor to determine the principal molecular/circuit targets of vortioxetine in adults with MDD, and possible additive or synergistic effects when combined with behavioral strategies (e.g. cognitive remediation) as well as assessing the effect of vortioxetine on cognitive function in the absence of depressive symptoms (e.g. residual cognitive symptoms in remitted patients).

## Supplementary Material

Supplementary Material Supplementary information supplied by authors.

Supplementary Material Supplementary information supplied by authors.

## Statement of Interest

**Sponsor:** H. Lundbeck A/S sponsored this study as part of a joint clinical development program with the Takeda Pharmaceutical Company, Ltd. www.ClinicalTrials.gov: Efficacy study of vortioxetine on cognitive dysfunction in adult patients with major depressive disorder (FOCUS); NCT01422213, http://clinicaltrials.gov/ct2/show/NCT01422213?term=NCT01422213&rank=1. Lundbeck was involved in the study design, in the collection, analysis and interpretation of data, in the writing of the report, and in the decision to submit the paper for publication. In the last 36 months Dr RS McIntyre has received research grant support from Lundbeck, Astra Zeneca, Pfizer, Shire, Otsuka, Bristol Myers Squibb, National Institute of Mental Health, Stanley Medical Research Institute, Canadian Institutes for Health Research, and The Brain and Behavior Research Foundation. Dr RS McIntyre has also received speaker/consultant fees from Lundbeck, Pfizer, Astra Zeneca, Elli Lilly, Janssen Ortho, Sunovion, Takeda, Forest, Otsuka, Bristol Myers Squibb and Shire. Drs Lophaven and Olsen are employees of H. Lundbeck A/S, the manufacturer of vortioxetine.

## Supplementary material

For supplementary material accompanying this paper, visit http://dx.doi.org/10.1017/S1461145714000546.
