# Supplementary material for: A randomized, double-blind, placebo-controlled study of vortioxetine on cognitive function in depressed adults
Source: Int J Neuropsychopharmacol. 2014 Apr 30;17(10):1557–67. doi: 10.1017/S1461145714000546 (PMC4162519; doi:10.1017/S1461145714000546)
Supplement: Supplementary Material — Supplementary information supplied by authors. [file S1461145714000546sup001.pptx]

## Slide 1
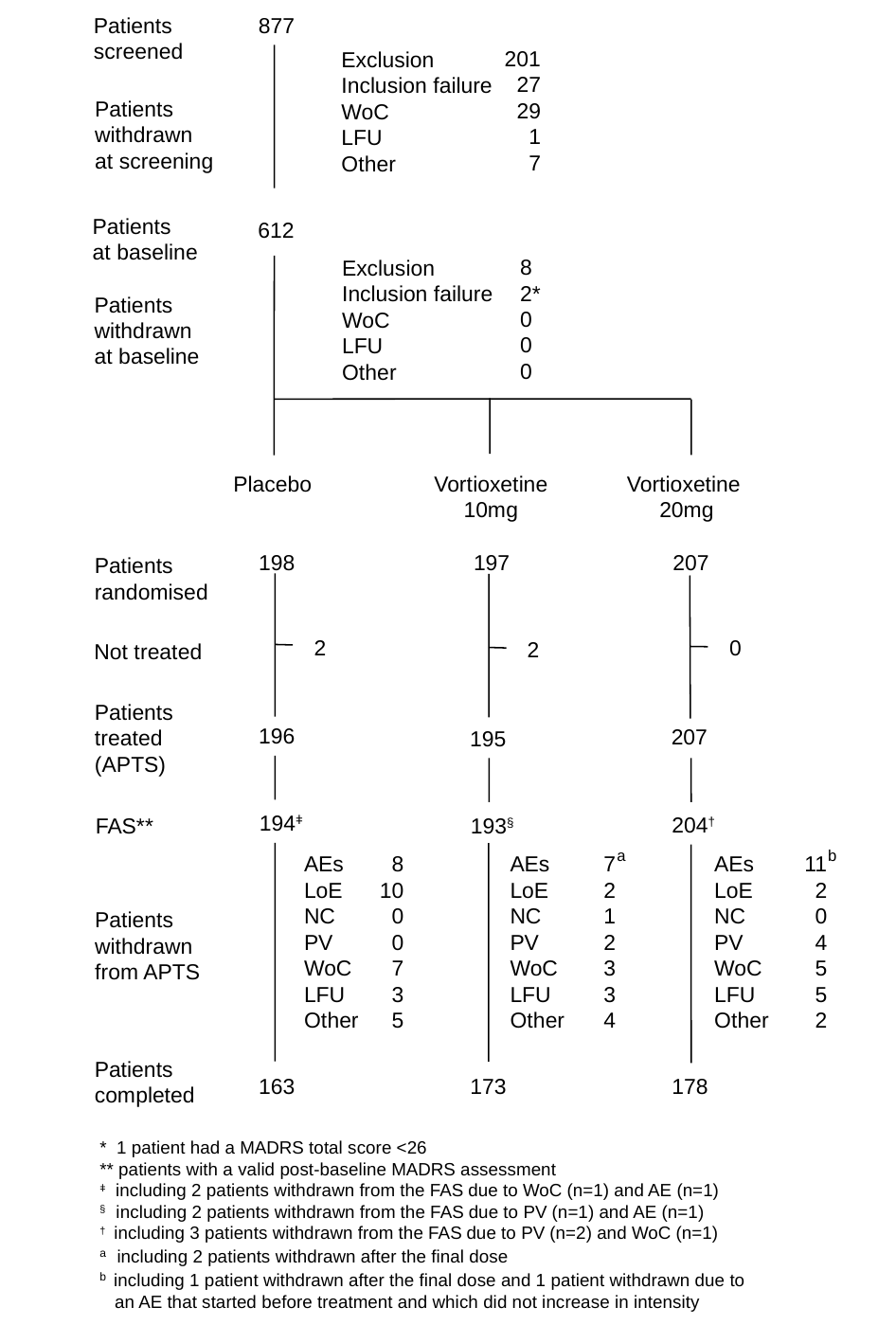

877
Patients
screened
201
27
29
1
7
Exclusion
Inclusion failure
WoC
LFU
Other
Patients
withdrawn
at screening
Patients
at baseline
612
8
2*
0
0
0
Exclusion
Inclusion failure
WoC
LFU
Other
Patients
withdrawn
at baseline
Placebo
Vortioxetine
10mg
Vortioxetine
20mg
198
197
207
Patients
randomised
2
0
2
Not treated
Patients
treated
(APTS)
196
207
195
194ǂ
204†
193§
FAS**
a
b
AEs
LoE
NC
PV
WoC
LFU
Other
8
10
0
0
7
3
5
AEs
LoE
NC
PV
WoC
LFU
Other
7
2
1
2
3
3
4
AEs
LoE
NC
PV
WoC
LFU
Other
11
2
0
4
5
5
2
Patients
withdrawn
from APTS
Patients
completed
163
173
178
* 1 patient had a MADRS total score <26
** patients with a valid post-baseline MADRS assessment
ǂ including 2 patients withdrawn from the FAS due to WoC (n=1) and AE (n=1)
§ including 2 patients withdrawn from the FAS due to PV (n=1) and AE (n=1)
† including 3 patients withdrawn from the FAS due to PV (n=2) and WoC (n=1)
a including 2 patients withdrawn after the final dose
b including 1 patient withdrawn after the final dose and 1 patient withdrawn due to
 an AE that started before treatment and which did not increase in intensity
