# Supplementary material for: A randomized, double-blind, placebo-controlled study of vortioxetine on cognitive function in depressed adults
Source: Int J Neuropsychopharmacol. 2014 Apr 30;17(10):1557–67. doi: 10.1017/S1461145714000546 (PMC4162519; doi:10.1017/S1461145714000546)
Supplement: Supplementary Material — Supplementary information supplied by authors. [file S1461145714000546sup002.docx]

**Contents:**

1. Additional information on Methods
   1. Patient exclusion criteria
   2. Description of assessment scales
   3. Primary efficacy analysis
   4. Key secondary efficacy analyses
   5. Secondary efficacy analyses
   6. Depressive symptoms efficacy analysis
   7. Direct effect on cognitive dysfunction
   8. Path analysis
2. Results: Figure S1: Flow chart of patient disposition
3. References

**Supplemental Material**

**Supplemental Methods**

*Patient exclusion criteria*

Patients were excluded if they had any current Axis I disorder other than MDD as defined in the DSM-IV-TR and confirmed using the MINI (Lecrubier et al., 1997), or a history of a manic or hypomanic episode, schizophrenia or any other psychotic disorder, mental retardation, organic mental disorders or mental disorders due to a general medical condition, any current diagnosis or history of substance abuse or dependence as defined in DSM-IV-TR, or the presence or history of a clinically significant neurological disorder, any neurodegenerative disorder, or any Axis II disorder that might compromise their participation in the study. The MINI was used to assess whether the patients had any other DSM-IV disorders, such as: major depressive episode with or without melancholic features, dysthymia, suicidality, (hypo)manic episode, panic disorder, agoraphobia, social phobia, obsessive-compulsive disorder, post-traumatic stress disorder, alcohol abuse and dependence, non-alcohol psychoactive substance abuse and dependence, psychotic disorders, anorexia nervosa, bulimia nervosa, generalized anxiety disorder, and antisocial personality disorder. Patients previously exposed to vortioxetine or with a score ≥70 on the Digit Symbol Substitution Test (DSST) (correct symbols), or ≥42 on the Rey Auditory Verbal Learning Test (RAVLT) (learning) or ≥14 on the RAVLT (memory) (Rey, 1964) at baseline, or diagnosed with dyslexia, were also excluded. Patients suffering from personality disorders, mental retardation, pervasive development disorder, attention-deficit/hyperactivity disorder, organic mental disorders, or mental disorders due to a general medical condition and patients with a diagnosis of alcohol or other substance abuse or dependence (excluding nicotine or caffeine) that had not been in sustained full remission at least 2 years prior to screening, physical, cognitive, or language impairment of such severity as to adversely affect the validity of the data derived from the neuropsychological tests, were not eligible.

Patients at serious risk of suicide, based on the investigator’s clinical judgment, or those who had a score ≥5 on item 10 of the Montgomery-Åsberg Depression Rating Scale (MADRS) (Montgomery and Åsberg, 1979) (*suicidal thoughts*) were excluded, as were those receiving formal psychological treatments; pregnant or breast-feeding women; those with current depressive symptoms considered by the investigator to have been resistant to two adequate antidepressant treatments of at least 6-weeks’ duration; or those who had previously been exposed to vortioxetine. Patients were also excluded if they were taking disallowed concomitant medication, as described by Alvarez et al. (2012) as well as the antibiotics rifampicin and ciprofloxacin, although anti-arrhythmics, anti-hypertensives (except metoprolol, carvediol, timolol and Class 1C anti-arrhythmics) and proton pump inhibitors (except omeprazole and cimetidine) were permitted. Episodic use of zolpidem, zopiclone or zaleplon for severe insomnia was allowed for a maximum of 2 days per week, but not the night before a study visit.

Patients were also excluded if they had a clinically significant unstable illness, a thyroid-stimulating hormone value outside the reference range and deemed clinically significant at screening, a history of cancer in remission for <5 years, or clinically significant abnormal vital signs as determined by the investigator. Patients with an abnormal ECG at screening considered by the investigator to be clinically significant, or a PR interval >250ms, a QRS interval >130ms, or a QTcF interval >450ms (for men) or >470ms (for women) were also excluded.

Safety reasons for withdrawal from the study were defined using the criteria described in Baldwin et al. (2012). In addition patients with a QTcF interval >500ms confirmed by ECG within 2 weeks and alanine aminotransferase/aspartate aminotransferase values outside pre-defined ranges were excluded. If adverse events (AEs) contributed to withdrawal, they were regarded as the primary reason for withdrawal.

*Description of assessment scales*

The DSST is an attention-demanding component of the Wechsler Adult Intelligence Scale (Wechsler, 1997). The DSST score was the number of digits coded correctly in a 90-second test period. The RAVLT assesses verbal learning and memory, including immediate memory, efficiency of learning, retroactive and proactive interference effects, and encoding versus retrieval (Rey, 1964; Lezak, 1983). The patient was given three trials to learn a list of 15 common nouns, and the acquisition score was the average number of words correctly recalled. The delayed recall score was the number of words correctly recalled after the other cognitive tests had been administered.

The Trail Making Test (TMT) assesses scanning, visuo-motor tracking, executive function, and cognitive flexibility (Lezak, 1983). The TMT consists of two parts: the patient connects consecutively numbered circles (part A) and then connects consecutively numbered and lettered circles, alternating between the two sequences (part B). The time taken to complete the two parts was recorded. The Stroop assesses the ability to inhibit a prepotent response to reading words while performing a task that requires attention control (Stroop, 1935). The Stroop test comprises two sheets with 50 words on each, and each word is the name of a color. On the first sheet, the Congruent Stroop Sheet, the word and ink color match; on the Incongruent Stroop Sheet, the word and ink color do not match. For each sheet, the patient has 4 minutes to name the ink color of each word. The time taken and the number of correct and incorrect responses were recorded.

The simple reaction time task (SRT) assesses psychomotor speed and the choice reaction time task (CRT) assesses visual attention. Two computerized tests, part of the CogState battery (Collie et al., 2007) were used to measure SRT and CRT (in ms). For SRT, the patient presses a "yes" button, whenever an onscreen playing card is turned over. For CRT, the patient presses a "yes" button whenever an onscreen playing card is turned over and is red, or a "no" button if the overturned card is not red.

The Perceived Deficits Questionnaire (PDQ) is a patient-rated scale designed to assess cognitive impairment/dysfunction that was administered after the neuropsychological tests and before the MADRS. The PDQ consists of four 5-item subscales: Attention/Concentration, Retrospective memory, Prospective memory and Planning/Organization (Sullivan et al., 1990). Each item is rated on a scale from 0 (never) to 4 (almost always). The total score of the 20 items ranges from 0 to 80, with higher scores reflecting greater subjective cognitive impairment.

*Primary efficacy analysis*

A statistical testing strategy was defined *a priori* and comprised the primary efficacy analysis as well as the key secondary efficacy analyses. To adjust for multiplicity, the 10 and 20mg doses of vortioxetine were tested separately *versus* placebo in the primary and key secondary efficacy analyses at a Bonferroni-corrected significance level of 0.05/2 = 0.025. The following sequence of hierarchically ordered primary and key secondary endpoints was used: difference in change from baseline to Week 8 between vortioxetine and placebo in:

1. the weighted sum of the z-scores in the DSST and RAVLT (primary)
2. the DSST (number of correct symbols)
3. the RAVLT (acquisition)
4. the RAVLT (delayed recall).

As soon as a hypothesis was rejected (that is, there was no statistically significant difference *versus* placebo at the 0.025 level of significance within a dose [10 or 20mg]), the testing procedure was stopped. For endpoints that occurred after the pre-specified statistical testing procedure was stopped or were outside the testing procedure, nominal p-values with no adjustment for multiplicity were reported. The phrasing ‘separation from placebo’ is used to describe findings with nominal p-values less than 0.05. Efficacy analyses that were not multiplicity-controlled were considered secondary. The principal statistical software used was SAS®, Version 9.2.

*Key secondary efficacy analyses*

The analyses of the key secondary continuous endpoints (DSST and RAVLT) were performed using the same methodology as for the primary efficacy analysis (full-analysis set [FAS], mixed model for repeated measures [MMRM]).

*Secondary efficacy analyses*

Cognitive dysfunction was measured as the difference in change from baseline to Week 8 between vortioxetine and placebo using the TMT-A, the TMT-B, the CRT, the Stroop test and the SRT. The changes from baseline to Week 8 in PDQ total and subscale scores were analyzed using an analysis of covariance (ANCOVA) model in which grouped site and treatment were included as factors and the baseline score was included as a covariate.

*Depressive symptoms efficacy analysis*

Depressive symptoms were measured as the change from baseline to Week 8 in the MADRS total score and the CGI-S (Guy, 1976) score using estimates from an MMRM model that was similar to the model specified for the primary endpoint. At Week 8, the following rates were calculated: response (defined as ≥50% decrease in MADRS total score from baseline), remission (defined as a MADRS total score ≤10), and CGI-I score. For analyses of the CGI-I score, the CGI-S score served as baseline. For dichotomous outcomes, such as response and remission, the primary methodology for analysis at Week 8 was logistic regression with treatment as factor and baseline score as a covariate.

In *post-hoc* analyses, Cohen's *d* was calculated for the neuropsychological tests as the difference between two means (vortioxetine and placebo) divided by the standard deviation for the pooled data (Cohen, 1988).

*Direct effect on cognitive dysfunction*

To evaluate whether the effect of vortioxetine on cognitive dysfunction was independent of depressive symptoms, the primary and DSST (number of correct symbols) analyses were repeated in a *post-hoc* analysis of patients who had not responded (<50% reduction from baseline in MADRS total score) and in patients who had not remitted (MADRS total score >10) by Week 8.

*Path analysis*

The proportion of cognitive dysfunction that was directly mediated, rather than indirectly mediated via depressive symptom improvement, was estimated using a protocol-specified path analysis (Ditlevsen et al., 2005). The effects were estimated using two ANCOVA models. In the first ANCOVA model, cognitive dysfunction is the outcome measured by the change from baseline in composite z-score at Week 8. Baseline composite z-score, baseline MADRS total score, treatment, site, baseline MADRS total score and change from baseline in MADRS total score were included as factors or covariates. The second ANCOVA is a model for the improvement in depression. In this model, change from baseline MADRS total score at Week 8 is the outcome while treatment, baseline MADRS and site are included as covariates or factors. The total treatment effect was determined as the estimated treatment effect in the first model plus the estimated treatment effect in the second model multiplied by the effect of an improvement in depression estimated in the first model. The direct treatment effect is then the treatment estimate in the first ANCOVA model. The indirect effect is the treatment effect estimate in the second ANCOVA model multiplied by the depression effect in the first model. The proportion of the treatment effect that is mediated through depression is then the proportion of the total effect that is ascribed to the indirect effect estimates. In this analysis, the two active treatment arms were pooled in one active arm; thus, in these analyses the significance level was 0.05.

**Figure 1.** Flow Chart of Patient Disposition. AEs: adverse events, LoE: lack of efficacy, NC: non-compliance, PV: protocol violation, WoC: patient consent withdrawn, LFU: lost to follow-up, MADRS: Montgomery-Åsberg Depression Rating Scale, BL: baseline, APTS: all-patients-treated set, FAS: full-analysis set.

**References**

Alvarez E, Perez V, Dragheim M, Loft H, Artigas F (2012) A double-blind, randomized, placebo-controlled, active-reference study of Lu AA21004 in patients with major depressive disorder (MDD). Int J Neuropsychopharmacol 15:589-600

American Psychiatric Association (APA) (2000) Diagnostic and Statistical Manual of Mental Disorders. Fourth Ed., Text Revision (DSM-IV-TR). Washington DC: American Psychiatric Association;

Baldwin DS, Loft H, Dragheim M (2012) A randomised, double-blind, placebo controlled, duloxetine-referenced, fixed-dose study of three dosages of Lu AA21004 in acute treatment of major depressive disorder (MDD). Eur Neuropsychopharmacol 22:482-491.

Cohen, J (1988) Statistical Power Analysis for the Behavioral Sciences. Second Edition. Hillsdale, NJ: Lawrence Erlbaum Associates, Publishers.

Collie A, Darekar A, Weissgerber G, Toh MK, Snyder PJ, Maruff P, Huggins JP (2007) Cognitive testing in early-phase clinical trials: development of a rapid computerized test battery and application in a simulated Phase I study. Contemp Clin Trials 28:391-400.

Ditlevsen S, Christensen U, Lynch J, Damsgaard MT, Keiding N (2005) the mediation proportion: a structural equation approach for estimating the proportion of exposure effect on outcome explained by an intermediate variable. Epidemiology 16:114-120.

Guy W, editor (1976) ECDEU Assessment Manual for Psychopharmacology. Revised Edition. Rockville, MD: National Institute of Mental Health.

Lecrubier Y, Sheehan DV, Weiller E, Amorim P, Bonora I, Sheehan KH, Janavs J, Dunbar GC (1997) The Mini International Neuropsychiatric Interview (MINI). A short diagnostic structured interview: reliability and validity according to the CIDI. Eur Psychiatry 12:224-231.

Lezak MD (1983) Neuropsychological Assessments. Second Edition. New York, NY. Oxford University Press.

Montgomery S, Åsberg M (1979) A new depression scale designed to be sensitive to change. Br J Psychiatry 134:382-389.

Rey A (1964) L‘examen clinique en psychologie [Clinical tests in psychology]. Paris, Presses Universitaires de France.

Sullivan JJL, Edgley K, Dehoux E (1990) A survey of multiple sclerosis Part 1: Perceived cognitive problems and compensatory strategy use. Can J Rehabil 4:99-105.

Stroop JR (1935) Studies of interference in serial verbal reactions. J Exp Psych 18:643-662.

Wechsler D (1997) Wechsler Adult Intelligence Scale. Third Edition. San Antonio, TX, Psychological Corporation.
